# Candida: The Ruthless Opportunist

**DOI:** 10.7759/cureus.37969

**Published:** 2023-04-22

**Authors:** Diogo Rebolo, Pedro Ventura, Mária Holgado, Vasco Neves, Luísa Lopes

**Affiliations:** 1 Intensive Care Medicine, Unidade de Saúde Local da Guarda, Hospital Sousa Martins, Funchal, PRT; 2 Internal Medicine Service, Unidade de Saúde Local da Guarda, Hospital Sousa Martins, Pinhel, PRT; 3 Intensive Care Medicine, Unidade de Saúde Local da Guarda, Hospital Sousa Martins, Guarda, PRT; 4 Internal Medicine Service, Unidade de Saúde Local da Guarda, Hospital Sousa Martins, Covilhã, PRT

**Keywords:** candida infections, candida, magnetic resonance imaging, antifungal, endoscopic retrograde cholangiopancreatography, sepsis, lithiasic pancreatitis, fungi, spondylodiscitis

## Abstract

Spondylodiscitis is a pathology with a devastating potential for functional limitation in patients, which may involve immobilization for months due to the risk of compression or even spinal cord section. It is a rare type of infection occurring in the vertebrae and discs of the spine, and most are bacterial. Fungal cases are rare.

We present the clinical case of a 52-year-old female patient with a past medical history of vesicular lithiasis and degenerative disc disease of the cervical spine and no home medication. The patient was hospitalized in the surgery service for about 3.5 months due to necro-hemorrhagic lithiasic pancreatitis that evolved into septic shock and needed organ support in intensive care for 2.5 weeks. Several cycles of antibiotics and endoscopic retrograde cholangiopancreatography (ERCP) with stent placement were performed. She was readmitted for urgent care to the hospital of residence with fever, sweating, and low back pain with sciatica five days after discharge. Lumbar CT and MRI evidence showed the destruction of about two-thirds of the vertebral bodies L3-L4, L5-S1, and adjacent discs, pointing to the diagnosis of infectious spondylodiscitis. Candida albicans was found in blood cultures and lumbar biopsies. The patient was treated with oral fluconazole 400 mg/day for eight months, and the control MRIs showed slow but favorable bone sclerosis over time. She spent a total of 13.5 months in the hospital, including five months in bedbound status. The patient left the hospital walking without any assistance, with an upright mood and disposition. The most likely main fungal infectious factors were the manipulation of the bile ducts, immunosuppression associated with corticosteroid therapy, and multiorgan septic failure.

The authors highlight this clinical case for its rarity, complications leading to candidemia, diagnostic and therapeutic delay, complexity, and risk of irreversible injuries to which the patient was subjected. The total recuperation of the patient after such a long physical and emotional struggle was very gratifying.

## Introduction

Vertebral osteomyelitis with discitis, or simply spondylodiscitis, is a pathology with a devastating potential for functional limitation in patients, which may imply immobilization for months due to the risk of compression or even medullary section [1]. It is rare, occurring in 2.9 to 5.4 per 100,000 inhabitants, and is responsible for about 1% of bone infections. It is twice as frequent in males, and its incidence increases with age (mostly over 50 years) [1, 2]. Currently, mortality due to this pathology is below 5%, and neurological sequelae are seen in less than 7% of survivors [3].

There are several risk factors for this pathology, such as endocarditis, valve prostheses, diabetes mellitus, degenerative pathology of the spine, use of injectable drugs, immunocompromised states, and, increasingly, intravascular devices and surgical and invasive procedures, such as endoscopic retrograde cholangiopancreatography (ERCP) in this case [2, 4-7]. The most common form of dissemination of the infection is hematogenous and may also be contiguous spread through trauma, invasive spinal procedures, or adjacent soft tissues with several types of abscesses, namely epidural, subdural, paraspinal, retropharyngeal, subphrenic, mediastinal, retroperitoneal, or psoas [2, 8, 9]. The starting point can even be meningitis or empyema [3]. In many cases, the reason and route of dissemination are unknown [4, 10-12].

Staphylococcus aureus is the agent involved in more than 50% of cases. Enteric Gram-negative bacilli, pyogenic and non-pyogenic Streptococcus, Pseudomonas aeruginosa, tuberculosis, and brucellosis may also be involved [7, 13, 14]. In rare cases, in about 0.5 to 1.6% of the total spondylodiscitis cases, the etiology is a fungus. Most of these are Candida species [2, 5, 8].

The main clinical manifestation is insidious and progressive osteoarticular pain in the affected disc and vertebral area, which worsens over days, weeks, and months with movement. This pain may radiate to the abdomen, perineum, legs, or scrotum [5, 15]. Fever is a finding that occurs in about 50% of cases [5, 16]. The development of a globus bladder is one of the plausible consequences of spinal cord compression, and pain in one of the flanks with hip extension reflects the possibility of a psoas abscess.

The gold-standard method for diagnosing this pathology is the isolation of a microorganism in the bacteriological or mycological culture of the affected tissue with a CT-guided biopsy. In patients with clinical, imaging, and analytical criteria that suggest the presence of this infectious pathology, the isolation in the blood culture of a plausible microorganism may be sufficient for the diagnosis [2, 7].

The treatment is the administration of antimicrobials directed at the microorganism in question, such as antibiotics or antifungals. These patients usually need multimodal analgesia [2, 13, 15, 16].

## Case presentation

This is the case of a 52-year-old woman with a past medical history of vesicular lithiasis and degenerative discopathy of the cervical spine who was hospitalized for 3.5 months in the general surgery service for necrohemorrhagic lithiasic pancreatitis complicated with peripancreatic collections and cholangitis, leading to multiorgan failure requiring intensive care for 2.5 weeks. There was a need for respiratory support with non-invasive mechanical ventilation (NIMV), renal replacement therapy (RRT), aminergic, and corticosteroid therapy. Multiple antimicrobial regimens were completed in the following sequence: piperacillin/tazobactam for eight days, meropenem for 17 days, vancomycin for nine days, piperacillin/tazobactam again for eight days, anidulafungin for five days, micafungin for 10 days, fluconazole for 14 days, metronidazole for 10 days, and linezolid for 10 days. This therapy was performed empirically due to infectious complications of pancreatitis and then directed therapy due to the positivity of blood cultures: a peripheral Staphylococcus epidermidis and a central and peripheral Candida albicans. During these antimicrobial cycles, there was a need for the placement of a biliary stent by ERCP. She was diagnosed with thrombosis of the superior vena cava nearly two weeks after ERCP, with an indication of three months of therapeutic anticoagulation.

**Figure 1:**
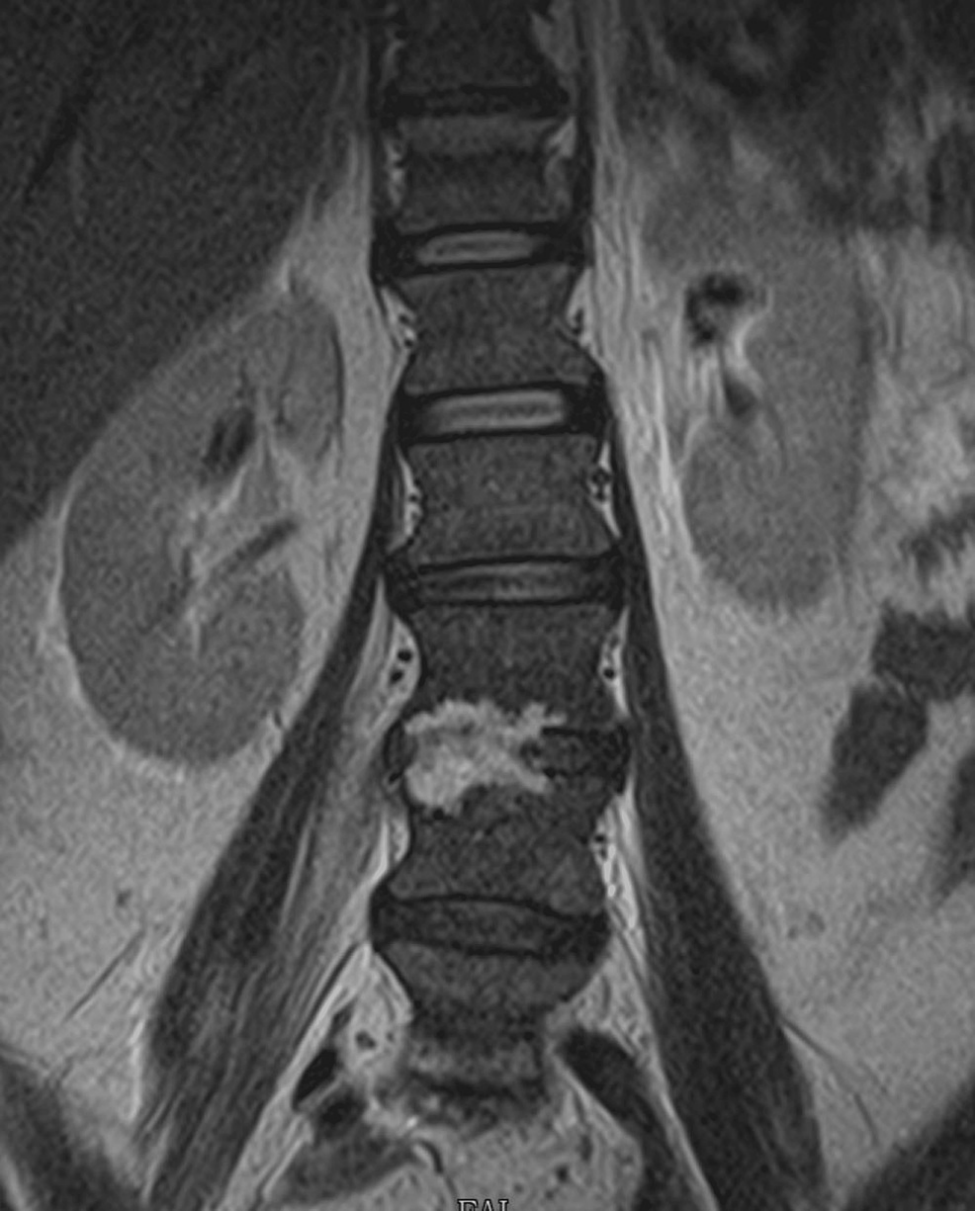
Magnetic resonance imaging (MRI) right before the beginning of the treatment (coronal view) Exuberant osteomyelitis is observed in the vertebral bodies of L3 and L4, but also in L5 and S1. Discitis is present between these vertebrae.

**Figure 2:**
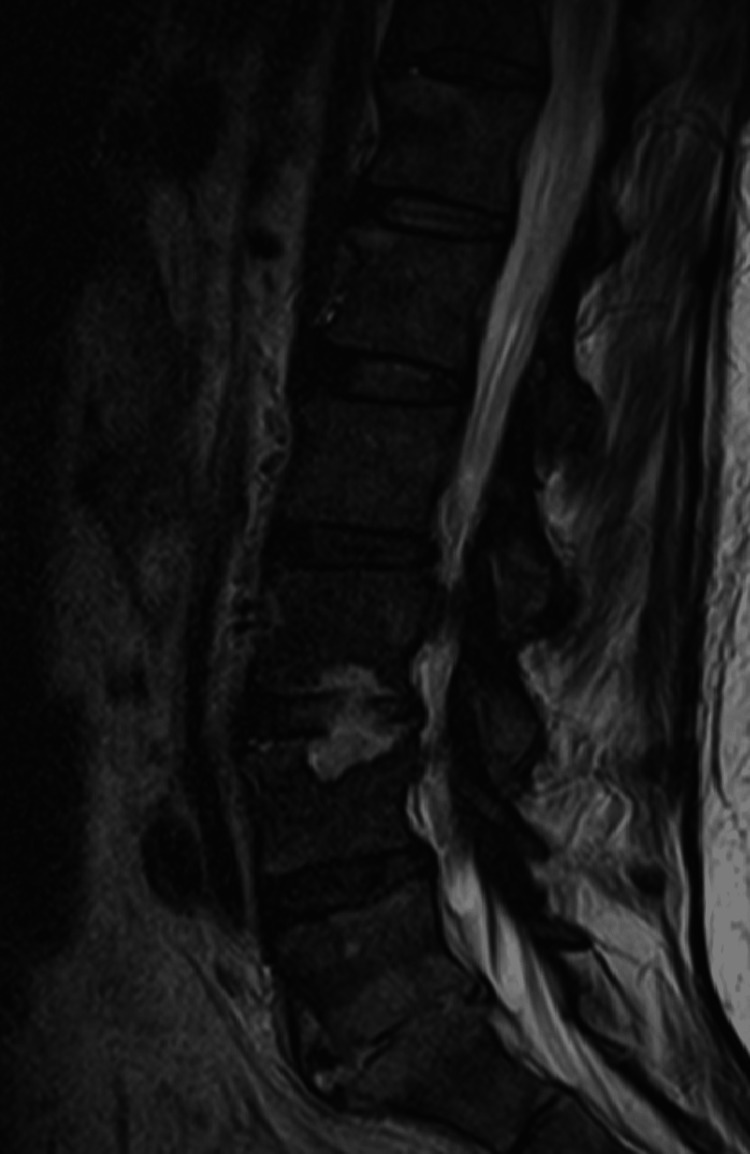
MRI right before the beginning of the treatment (sagittal view)

Only four days after hospital discharge, the patient was readmitted to the emergency department due to sudden and rapidly progressive severe low back pain with irradiation to both lower limbs in the form of sciatica, accompanied by fever (>38.5ºC) and sweating. After computed tomography (CT) of the lumbar spine, she was hospitalized with suspected infectious spondylodiscitis. Magnetic resonance imaging (MRI) showed the destruction of about two-thirds of the vertebral bodies L3, L4, L5, S1, and adjacent discs. (Figures 1-2).

Candida albicans was again isolated in peripheral blood culture, the same agent that would later be identified in a CT-guided biopsy of the lesion. The diagnosis of fungal spondylodiscitis was made. She was managed by general surgery, orthopedics, infectious diseases, physical therapy, and neurosurgery. Surgical intervention was deferred due to imageological and clinical neurological stability.

**Figure 3:**
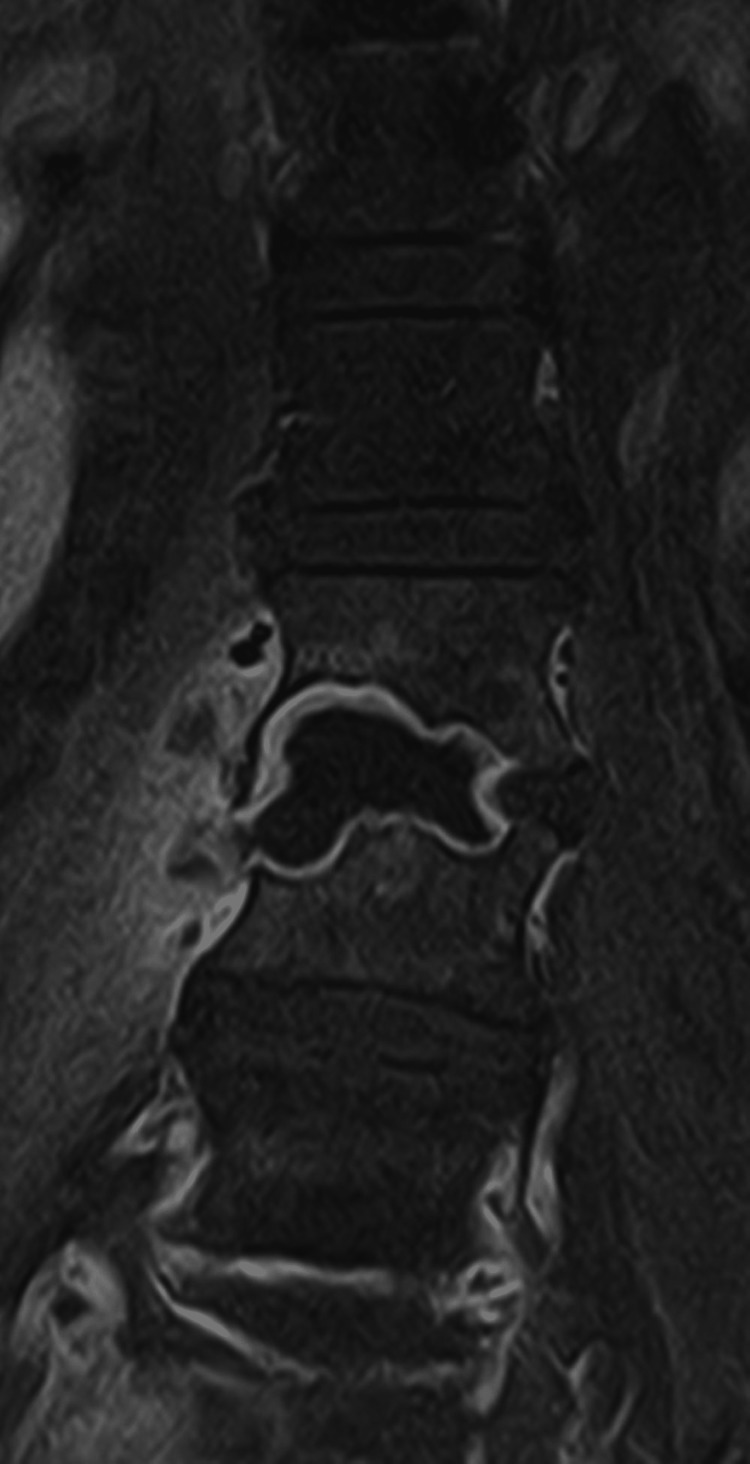
MRI six weeks after the start of the treatment (coronal view).

**Figure 4:**
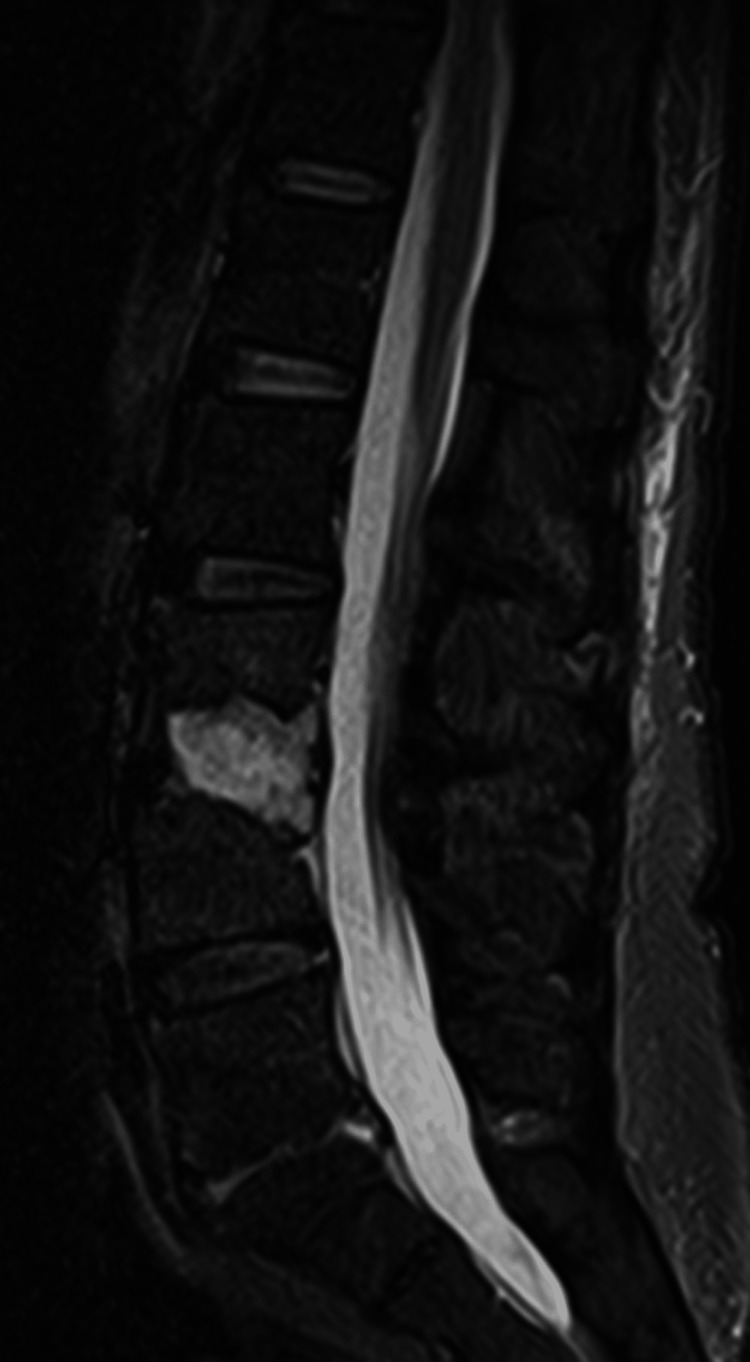
MRI six weeks after the start of the treatment (sagittal view).

**Figure 5:**
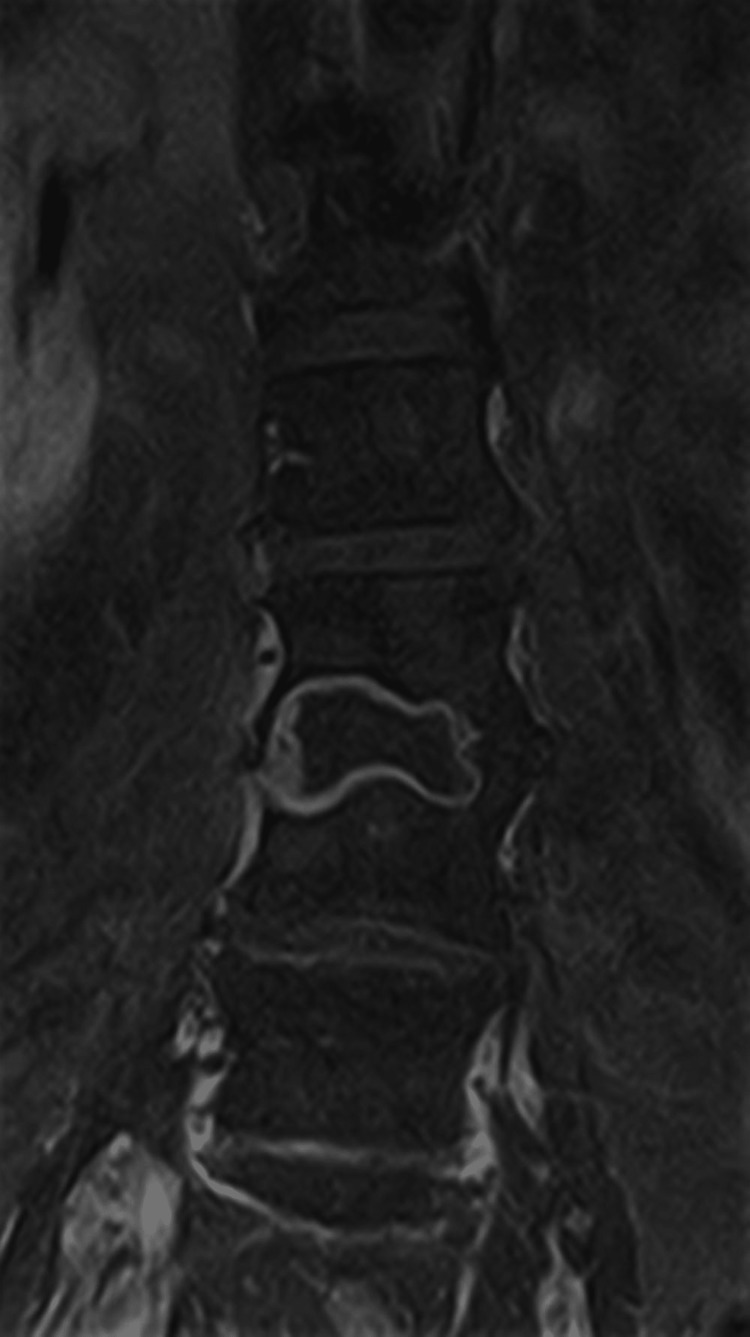
Last control MRI, 11 months after the beginning of the treatment (coronal view)

**Figure 6:**
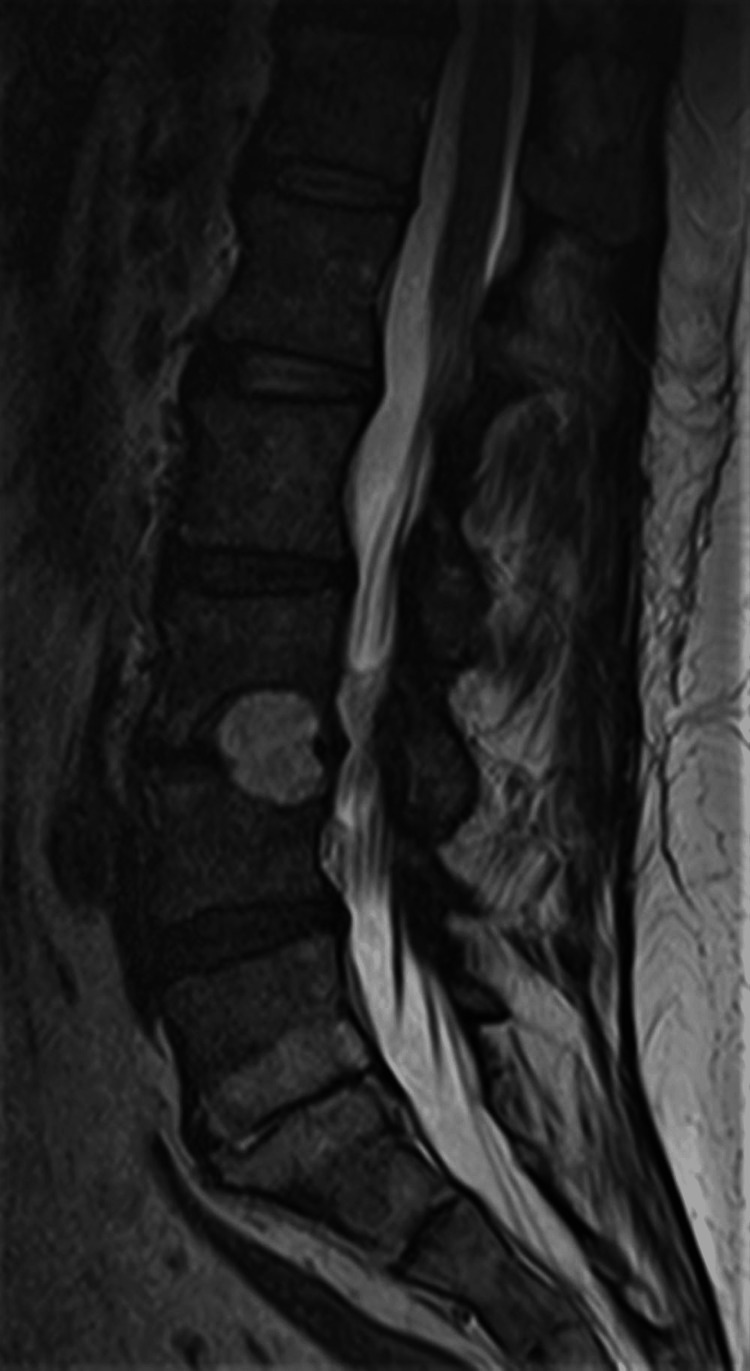
Last control MRI, 11 months after the beginning of the treatment (sagittal view)

The patient was transferred to the internal medicine ward. We started direct therapy with micafungin for 14 days, followed by oral fluconazole 400 mg/day for eight months, as recommended by infectious disease colleagues. She was administered multimodal analgesia with paracetamol, metamizole, tramadol, gabapentin, and SOS non-steroidal anti-inflammatory drugs (NSAIDs). The patient showed a clinically slow but sustainably favorable evolution. A follow-up MRI at six weeks after therapeutic onset demonstrated a marked reduction of inflammatory tissue and an increased area of bone sclerosis, which continued in the next few months in control MRIs (Figures 3-6).

Physiotherapy was performed during this process. Collaboration with psychiatry was necessary for depressive assessment. 

In the order of this evolution, seven months after the first admission due to pancreatitis, the patient started mobilization with a lift using a lumbostat, with a formal neurosurgical indication. She was hospitalized for around 13.5 months, five of which were spent in a supine position without going through an orthostatic or sitting position. The patient left the hospital walking without any help, with her mood and disposition intact.

## Discussion

The demonstration of the present clinical case aims to evidence the rarity and possible severity of fungal spondylodiscitis [1, 7]. Usually, this pathology has some delay in definitive diagnosis due to the procedures inherent to this purpose, requiring CT-guided biopsy in most cases. The symptomatology of thoracic or lumbar spine pain is a predictor of multiple differential diagnoses, going through the various pathologies of the spine, including arthrosis and arthritis, discopathies, abscessed formations, root compressions for different reasons, and muscle or organ pathologies [2]. Commonly, spondylodiscitis pain is insidious in onset, worsens over weeks or months at night, and may be absent in paraplegic patients. This risk of significant peripheral and/or central neurological alterations is higher in patients with the co-existence of abscessed formations in associated peri spondylodiscitis lesion zones [6]. Although fever has been found in this case, it is an inconstant clinical finding in this pathology, with frequencies up to 52% of cases and even less, as described in the scientific literature [7, 16].

It can be observed from this clinical case that the severity of this pathology is associated with drastic limitations in the patient's life, such as marked immobilization with bedbound status and only slight lateral mobilizations. The need to maintain this position is related to the possibility of partial or total vertebral fracture in an orthostatic or sitting position, with consequent compression or even medullary and/or root section [11,15]. This prolonged treatment duration is why there is a need for great precaution in mobilization. Meanwhile, eradicating osteomyelitis and discitis with antibiotics or antifungals directed at the microorganism concerned is a time-consuming process due to the type of tissue and the difficult penetration of antimicrobials. Calcification and bone sclerosis with new tissue formation also contribute to the need for prolonged immobilization as well as the indispensability of surgery in some cases, particularly in those with abscesses [17,18].

In this case, fluconazole therapy was required at a dose of 400 mg/day for eight months until there was the total elimination of the Candida albicans infection and sufficient bone formation for the safe adoption of the orthostatic position. As provided in this case, these patients require a lot of physiotherapy sessions to attenuate muscle atrophy caused by these long hospitalizations and the associated analgesia [6].

Although this patient had paravertebral abscesses adjacent to the bone and a discitis lesion, neurosurgery decided that the risks of surgical intervention would outweigh the benefits due to the stability of the lesion observed on MRI. The fact that there was no emerging neurological impairment also led to no surgical intervention.

Usually, these patients need multimodal analgesia, with analgesics for neuropathic pain, opioids, paracetamol, metamizole, SOS NSAIDs, or even epidural analgesia.

Even though one of the most prevalent etiologies of this pathology is associated with endocarditis and cardiac valve prostheses, in this case, it was inferred that the most probable cause was the manipulation of the bile ducts with the placement of a biliary stent by ERCP in association with multiorgan septic failure and corticosteroid therapy.

## Conclusions

Patients with this pathology require extreme care due to the risk of fractures and need multimodal analgesia for the pain they endure, along with physiotherapy. We highlight the patient's complete clinical, analytical, and imaging recovery, despite the serious risks she was exposed to during a very prolonged hospital stay. In addition to physiological treatment, these patients require psychological follow-up, taking into account the emotional struggle inherent in them.
